# Phosphoenolpyruvate Carboxykinase Controls *Edwardsiella tarda* Virulence via Oxaloacetate

**DOI:** 10.3390/biology15161338

**Published:** 2026-08-07

**Authors:** Jiayao Wu, Feirong Yuan, Zilong Huang, Sihui Huang, Tianshun Kou, Xuanwei Chen, Bo Peng

**Affiliations:** 1 State Key Laboratory of Biocontrol, Guangdong Key Laboratory of Pharmaceutical Functional Genes, School of Life Sciences, Southern Marine Science and Engineering Guangdong Laboratory (Zhuhai), Sun Yat-sen University, Guangzhou 510275, Chinayuanfr@mail2.sysu.edu.cn (F.Y.);; 2 Laboratory for Marine Biology and Biotechnology, Qingdao Marine Science and Technology Center, Qingdao 266071, China

**Keywords:** phosphoenolpyruvate carboxykinase, oxaloacetate, virulence, *Edwardsiella tarda*, auto-aggregation

## Abstract

Understanding the metabolic basis of bacterial virulence is essential for developing anti-infective strategies. Phosphoenolpyruvate carboxykinase (PEPCK), encoded by *pckA*, is a key enzyme at the metabolic junction between the pyruvate cycle and the TCA cycle, but its role in virulence regulation remains largely unexplored. In this study, we generated a *pckA* deletion mutant, *ΔpckA*, in *Edwardsiella tarda* and investigated its impact on virulence, metabolism, and vaccine potential. The *ΔpckA* mutant exhibited increased antibiotic sensitivity, faster growth, reduced autoaggregation, and significantly attenuated virulence in a tilapia infection model. Quantitative proteomics revealed global downregulation of type III and type VI secretion systems (T3SS and T6SS) and upregulation of flagellar proteins. Mechanistically, accumulation of oxaloacetate (OAA) in the mutant suppressed T3SS/T6SS expression without affecting flagellar motility. Importantly, *ΔpckA* induced a robust immune response in zebrafish and conferred strong protection against lethal challenge, with relative percent survival values of 64% and 81.82% in zebrafish and tilapia, respectively. Taken together, our findings identify PEPCK as a critical metabolic regulator of *E. tarda* virulence and highlight *ΔpckA* as a promising live attenuated vaccine candidate.

## Simple Summary

*Edwardsiella tarda* is a bacterium that causes severe disease in fish, leading to major economic losses for aquaculture worldwide. Understanding how this bacterium causes infection is essential for developing effective prevention strategies. In this study, we investigated a key bacterial enzyme called phosphoenolpyruvate carboxykinase (PEPCK), which helps control the bacterium’s energy metabolism. When we removed the gene responsible for producing this enzyme, the bacteria became less harmful to fish, grew faster, and showed increased movement. Importantly, this modified bacterium triggered a strong immune response in fish and protected them against subsequent infection with the deadly form of the bacteria. Fish that received the modified bacteria survived at much higher rates compared to unprotected fish. These findings reveal that this enzyme plays a critical role in controlling bacterial disease-causing ability through its effects on cellular metabolism. The modified bacteria show promise as a safe and effective vaccine candidate to protect farmed fish against *Edwardsiella* infections, potentially reducing disease outbreaks and economic losses in aquaculture while decreasing the need for antibiotic treatments.

## 1. Introduction

Understanding the interplay between antibiotic resistance and bacterial virulence is cruccial for addressing the threat posed by multidrug-resistant bacteria. While it was traditionally assumed that acquiring resistance comes at a biological cost (e.g., reduced growth or virulence), emerging evidence reveals a more complex picture that some resistance mechanisms are virtually cost-free, and in certain cases, they even enhance pathogenic potential [1]. For example, antibiotic-resistant bacteria can be less virulent but persist longer in the host than the sensitive counterpart [2]. Therefore, deciphering this balance is essential for predicting the spread of resistant strains in the absence of antibiotic pressure and for developing alternative therapeutic strategies, such as anti-virulence approaches or resistance-reversal therapies [3,4]. Given the extraordinary resistance capacity and virulence arsenal of the multidrug-resistant pathogens, such research holds significant clinical relevance [5,6,7].

Recent studies highlight that bacterial metabolism plays a key role in mediating the trade-off between resistance and virulence. For instance, hyperproduction of the AmpC β-lactamase affects peptidoglycan metabolism, leading to altered cell wall architecture and reduced motility and virulence [8,9,10]. Additionally, overexpression of efflux pumps such as MexEF-OprN triggers metabolic compensation, including upregulation of the anaerobic nitrate respiratory chain, which alleviates fitness costs and represents a potential therapeutic target [11,12,13]. However, cases in the mobile colistin determinant (*mcr-1*) gene is more complicated. While *mcr-1* generally imposes a fitness cost that tends to attenuate bacterial virulence, the outcome is not unidirectional and depends critically on the genetic background, plasmid type, co-resident resistance/virulence genes, and environmental selection pressures [14,15]. Thus, the perspective at genomic level may not be a comprehensive way understand the trade-off between antibiotic resistance and virulence.

Recent advances indicate that bacterial metabolism plays a pivotal role in shaping antibiotic susceptibility and the bactericidal outcome [16]. The metabolic state determines the antibiotic susceptibility and the rewiring metabolism could be a potential way to enhance antibiotic killing [17,18]. Seminal work has established that bactericidal antibiotics kill bacteria by perturbing the tricarboxylic acid (TCA) cycle and respiratory chain, thereby triggering the production of deleterious reactive oxygen species (ROS) [19,20]. Subsequent studies revealed that antibiotic-mediated disruption of the proton motive force (PMF) and cellular respiration directly influencing drug uptake, linking energy metabolism to intrinsic susceptibility [21]. Beyond these studies, exogenous metabolites, such as mannitol, have been shown to revive bacterial metabolism and effectively eliminate tolerant persisters [22]. Notably, beyong antibiotic tolerance, emerging evidence indicates that specific metabolites, such as pyruvate, formate, succinate, fumarate and methionine et al., are also shown their capability in reversing antibiotic resistance and potentiate antibiotic killing in various bacterial species [23,24,25,26,27], reinforcing the therapeutic promise of metabolic reprogramming. In the same time, metabolic remodeling not only influences growth rates but also modulates quorum sensing, toxin production, and biofilm formation, suggesting that targeting metabolic pathways could offer an unrecognized anti-infective strategies, reducing both the fitness benefits of resistance and bacterial virulence [28,29]. However, the molecular mechanisms by which metabolism coordinates antibiotic susceptibility and virulence remain largely unexplored, prompting our investigation into the role of PEPCK in *E. tarda*.

Phosphoenolpyruvate carboxykinase (PEPCK), encoded by *pckA*, is a key enzyme at the junction of the pyruvate cycle and the TCA cycle. It catalyzes the reversible conversion of oxaloacetate (OAA) to phosphoenolpyruvate (PEP), thereby influencing gluconeogenesis, anaplerosis, and overall metabolic flux [30]. In several pathogens, PEPCK has been implicated in intracellular survival and persistence [4], but its role in regulating bacterial virulence is poorly understood.

*Edwardsiella tarda* is a Gram-negative *Hafniaceae* family bacteria that causes severe hemorrhagic septicemia in fish, leading to substantial economic losses in aquaculture worldwide [31]. Its virulence relies on a complex arsenal of secretion systems, including type III and type VI secretion systems (T3SS and T6SS), which deliver effectors into host cells, as well as flagella-mediated motility that facilitates initial host colonization [32,33]. Additionally, auto-aggregation is a typical virulent feature of *E. tarda* [34]. Despite these known factors, the metabolic cues that coordinate their expression remain elusive.

Despite the recognized roles of T3SS, T6SS, and flagellar motility in *E. tarda* pathogenesis, the metabolic signals that coordinate their expression remain poorly defined. In this study, we investigated whether PEPCK, a key gluconeogenic enzyme at the pyruvate–TCA cycle junction, contributes to virulence regulation. By combining genetic deletion, quantitative proteomics, and phenotypic analyses, we aimed to uncover the metabolic basis of virulence control in *E. tarda* and to evaluate the vaccine potential of a *pckA* mutant.

## 2. Materials and Methods

### 2.1. Ethic Approval

The Institutional Animal Care and Use Committee of Sun Yat-sen University reviewed and approved all animal experiments in this study (Approval No. SYSU-IACUC-2020-B1267). All animal procedures strictly followed the ARRIVE 2.0 guidelines.

### 2.2. Bacterial Strains and Growth Condition

The *E. tarda* strain EIB202 employed in this study was originally isolated from a diseased turbot and kindly provided by Prof. X. H. Zhang at Ocean University of China as previously described [35]. Stock cultures were kept at −80 °C. To prepare the bacterial suspension, a single colony from a tryptic soy broth (TSB) agar plate was inoculated into 50 mL of TSB medium and incubated at 30 °C for 24 h on a rotary shaker at 200 rpm. The resulting culture was then diluted 100-fold with fresh TSB and grown further until the OD_600_ value reached 1.0. Bacterial cells were collected by centrifugation (6000 rpm, 5 min, 4 °C) and rinsed three times with sterile 0.85% saline. The final pellet was re-suspended in the same saline solution for subsequent infection experiments.

### 2.3. Construction of pckA Mutant

A previously reported one-step procedure was adopted to generate the *pckA* deletion mutant [36]. Based on the sequences of plasmid pKD13 and the *pckA* gene, primers *pckA*-F and *pckA*-R were designed. A kanamycin resistance cassette was amplified by PCR using pKD13 as the template. The purified PCR product was introduced into *E. tarda* EIB202 via electroporation; this strain had already been transformed with the temperature-sensitive plasmid pSIM6. Homologous recombination between the genomic *pckA* region and the amplified fragment was driven by the λRED recombinase encoded by pSIM6. Kanamycin selection was applied to isolate the mutant, and the precise deletion of *pckA* was verified through PCR and DNA sequencing. All primer sequences are provided in Table S2.

### 2.4. Growth Curve Measurement

Overnight cultures initiated from single colonies were diluted 1:1000 into fresh TSB medium and incubated at 30 °C with shaking. Bacterial growth was monitored by measuring OD_600_ every 2 h for 24 h. All experiments were conducted with three independent biological replicates.

### 2.5. Intracellular Oxaloacetate (OAA) Quantification

Intracellular OAA levels in the wild-type EIB202 and *ΔpckA* mutant strains were quantified using the Amplex Red Oxaloacetate Assay Kit (Beyotime Biotechnology, Shanghai, China). Briefly, bacterial strains were grown overnight to saturation in liquid medium at 30 °C with shaking. The cultures were harvested by centrifugation to remove the medium, washed once with phosphate buffer saline (PBS), and adjusted to a uniform initial optical density at 600 nm (OD_600_) of 1.0. For each sample, 1 mL of the adjusted bacterial suspension was centrifuged, and the cell pellet was resuspended in 100 μL of BeyoLysis™ Buffer A (Beyotime Biotechnology, Shanghai, China) for Metabolic Assay. After incubation on ice for 10 min to allow for complete lysis, cell debris was removed by centrifugation at 12,000× *g* for 5 min at 4 °C. The resulting supernatants were immediately collected for OAA measurement.

The assay was performed strictly following the manufacturer’s instructions. A standard curve was established using a series of OAA standard concentrations. In a microplate, 20 μL of each sample or standard was mixed with 80 μL of the enzymatic working solution. The reaction mixture was incubated at 37 °C for 60 min in the dark. Finally, the absorbance at 570 nm was recorded using a microplate reader. All experimental groups were tested with three technical replicates. The final OAA concentrations were calculated directly based on the standard curve.

### 2.6. Auto-Aggregation Assay

Aggregation assays were conducted following established procedures with minor modifications [37]. Briefly, single colonies were inoculated into Dulbecco’s modified Eagle medium (DMEM) or tryptic soy broth (TSB) and incubated at 30 °C; DMEM cultures were maintained in a 5% CO_2_ atmosphere, whereas TSB cultures were grown under static conditions. After 24 h, cultures were diluted 1:50 into fresh medium and transferred to transparent glass tubes, followed by incubation under the same respective conditions. Bacterial aggregation was assessed by recording images at 24 h after subculture.

To quantify the effect of oxaloacetate (OAA; Aladdin, Shanghai, China) on auto-aggrega tion, three independent single colonies were picked from the solid plates of both wild-type EIB202 and the *ΔpckA* mutant, and inoculated into six individual tubes containing 5 mL of TSB for initial overnight incubation at 30 °C with shaking to saturation. The overnight cultures were diluted 1:100 into fresh TSB. For EIB202, OAA was added to the subcultures at final concentrations of 0 (blank control), 0.5, 5, 25, and 50 mM. For the *ΔpckA* mutant, subcultures without OAA were used as a blank control. Following an overnight incubation with shaking, the cultures were allowed to stand statically overnight. Auto-aggregation was visually documented and quantitatively assessed by measuring the upper supernatant in a microplate reader (EPOCH, BioTek, Winooski, VT, USA) at OD_600_. Finally, the cultures were thoroughly homogenized, and the OD_600_ was re-measured to evaluate total bacterial growth.

### 2.7. Evaluation of Physical Viscosity on Bacterial Aggregation Using Glycerol as a Control

First, a preliminary experiment was conducted to screen for the appropriate glycerol concentration as a physical viscosity control. An overnight saturated culture of the wild-type strain EIB202 was subcultured into 50 mL of medium and grown to saturation. The culture was then aliquoted into centrifuge tubes (5 mL per tube). The experimental groups were supplemented with either 25 mM oxaloacetic acid (OAA) or a final concentration gradient of glycerol (0%, 0.05%, 0.1%, 1%, 2%, 5%, and 10%) and mixed thoroughly. To simulate the sedimentation process, the mixtures were centrifuged at 500× *g* for 15 min, followed by photographic documentation of the tubes. Subsequently, 100 μL of the supernatant from each tube was carefully transferred into a 96-well plate, and the OD_600_ was measured using a microplate reader. The extent of bacterial aggregation (indicated by the supernatant OD_600_ values) following centrifugation in the presence of 25 mM OAA was most comparable to that of the 0.1% glycerol group. Therefore, 0.1% glycerol was selected as the optimal physical viscosity control concentration for subsequent static aggregation assays.

Next, a formal static aggregation assay was performed. Five experimental groups were established: (1) EIB202 control; (2) EIB202 + 0.1% glycerol (glycerol was added after the culture reached saturation); (3) EIB202 + 25 mM OAA; (4) *ΔpckA* control; and (5) *ΔpckA* + 0.1% glycerol. Once the cultures reached saturation and the respective additions were completed as designed, they were mixed thoroughly and photographed to record the initial state. All tubes were then statically incubated at room temperature for 5 h to observe natural bacterial aggregation and sedimentation. After the static incubation, the tubes were photographed again, and the OD_600_ of the supernatant was measured using a spectrophotometer. Finally, all cultures were vigorously vortexed to a homogenous state, and the total OD_600_ was measured to verify that there were no significant differences in total biomass among the treatment groups.

### 2.8. Fish Acquisition and Husbandry

Zebrafish, *Danio rerio*, (mean weight 0.2 ± 0.05 g) and Nile tilapia, *Oreochromis niloticus*, (mean weight 20 ± 2.5 g) were obtained from the Guangdong Tilapia Breeding Farm (Guangzhou, China). Additional tilapia used for bacterial challenge experiments were purchased from a separate commercial farm in Guangdong Province, with verbal consent for research use secured from the farm owner prior to purchase. All fish were acclimated for two weeks in recirculating tanks (5 L for zebrafish, 120 L for tilapia) at 28 °C. Before any infection experiment, the absence of *Edwardsiella* contamination was confirmed via microbiological testing.

Prior to injection, tilapia were anesthetized by immersion in tricaine methanesulfonate (MS-222, 100 mg/L). At the experimental endpoint, euthanasia was performed with an overdose of MS-222 (300 mg/L) followed by cervical transection to ensure death, in accordance with the AVMA Guidelines for the Euthanasia of Animals (2020 edition) [2]. All procedures were designed to minimize suffering.

### 2.9. Bacterial Preparation and In Vivo Infection Assay

As described previously [25,38], bacteria were subcultured in TSB medium until the optical density at 600 nm (OD_600_) reached 0.7. The cell pellet was collected by centrifugation and resuspended in sterile 0.85% saline to an OD_600_ of 1.0. This suspension was further diluted to the desired concentrations for intraperitoneal injection.

To evaluate the virulence of the different strains, tilapia were randomly divided into ex perimental groups (*n* = 14 fish per group). Fish received an intraperitoneal injection of either the wild-type EIB202 or the *ΔpckA* mutant at a dose of 2 × 10^6^ CFU/fish. Mortality was monitored and recorded daily for 7 days post-infection.

### 2.10. Proteomics Sample Preparation

Proteomic analysis of bacteria was performed as previously described [39,40]. For each of the wild-type strain and the *ΔpckA* mutant, three independent single colonies were selected and each inoculated into 30 mL of tryptic soy broth (TSB, Huan Kai Microbial). The cultures were grown at 30 °C with orbital shaking at 180 rpm for approximately 23 h until they reached the stationary phase. Cells were then pelleted by centrifugation at 5000 rpm for 15 min at 4 °C, and the supernatant was removed. The resulting pellets were resuspended in ice-cold PBS and rinsed three times to eliminate any remaining medium components. After washing, the pellets were immediately frozen in liquid nitrogen and kept at −80 °C until protein extraction for subsequent proteomic analysis.

### 2.11. Mass Spectrometry Analysis

Mass spectrometry analysis was performed as previously described [41]. For every sample, 2 µg of total peptides were subjected to separation and analysis using nano-ultra-performance liquid chromatography on an EASY-nLC1200 system coupled to a Q-Exactive HFX Orbitrap mass spectrometer (Thermo Fisher Scientific, Waltham, MA, USA) fitted with a nano-electrospray ionization source. A reversed-phase column (EASY-Spray HPLC column, 150 µm internal diameter × 15 cm, Thermo Fisher Scientific, Waltham, MA, USA) was used for peptide separation. The mobile phase consisted of solvent A (0.1% formic acid in 2% acetonitrile) and solvent B (80% acetonitrile with 0.1% formic acid). A 90-min gradient at a flow rate of 300 nL/min was applied as follows: solvent B increased from 2% to 5% over 2 min, then from 5% to 22% over 68 min, followed by an increase to 45% over 16 min, then to 95% over 2 min, and finally held at 95% for another 2 min.

Data-dependent acquisition (DIA) was carried out using an Orbitrap analyzer operated in positive ion mode, with a resolution of 120,000 (at 200 m/z) for full MS1 scans across an m/z range of 350–1600. For MS2 scans, the resolution was set to 45,000 with a fixed first m/z of 110. The automatic gain control (AGC) target was 3 × 10^6^ for MS1 with a maximum injection time (IT) of 30 ms; for MS2, the AGC target was 1 × 10^5^ with a maximum IT of 96 ms. The top 20 most intense precursor ions were fragmented using higher-energy collisional dissociation (HCD) at a normalized collision energy (NCE) of 32% and an isolation window of 0.7 m/z. Ions with a single charge or charges greater than six were excluded from DDA, and the dynamic exclusion duration was set to 45 s.

### 2.12. DIA-NN Database Search

Raw MS data obtained from the vendor were processed using DIA-NN (version 2.2.0). The MS/MS spectra were queried against a species-specific UniProt FASTA database (uniprotkb_taxonomy_id_1263550_2025_08_20.fasta). Carbamidomethylation of cysteine was set as a fixed modification, whereas oxidation of methionine and N-terminal acetylation were selected as variable modifications. Trypsin was designated as the digestion enzyme, permitting up to two missed cleavages. The false discovery rate (FDR) was controlled at 0.01 for both peptide-spectrum matches (PSMs) and peptide identifications. Precursor and fragment mass tolerances were set to 20 ppm for peptide identification. All remaining parameters were kept at their default values.

### 2.13. Bacterial Motility Assay

Bacterial motility was evaluated following previously established protocols [42,43]. Briefly, overnight cultures were diluted 1:100 into fresh TSB medium and incubated until the OD_600_ reached 0.5. A 2-μL aliquot of the exponential-phase culture was spotted onto the center of TSB plates supplemented with either 0.3% or 0.6% (*w*/*v*) agarose. Following incubation, the radii of the motility halos were measured at designated time points.

### 2.14. Biofilm Formation Assay

Biofilm formation was evaluated using a modified crystal violet staining method [37,44]. Briefly, three independent single colonies of *E. tarda* strains were selected and cultured overnight in Tryptic Soy Broth (TSB) to serve as biological replicates. The overnight cultures were then diluted 1:50 into fresh DMEM and inoculated into 24-well culture plates equipped with glass coverslips. The plates were maintained in a CO_2_ incubator for 24 h. Subsequently, the planktonic cell supernatants were discarded, and the coverslips were gently washed three times with PBS to eliminate non-adherent bacteria. The established biofilms were fixed with 4% PFA and stained with 0.1% crystal violet for 30 min. After photographic documentation, the bound dye was solubilized with 200 μL of 1% (*w*/*v*) sodium dodecyl sulfate (SDS) buffer per well. The absorbance, reflecting biofilm biomass, was measured at 595 nm using an EPOCH microplate reader (BioTek). Each biological replicate was assessed in triplicate.

### 2.15. Quantitative Real-Time PCR (qRT-PCR)

Real-time PCR was employed to determine gene expression levels as previously described [36,45]. For bacterial samples, RNA was isolated using an RNA extraction kit (EZB biosciences, Rochester, MN, USA); for fish tissues (spleen and head kidney), TRIZOL reagent (Invitrogen Life Technologies, Carlsbad, CA, USA) was used. One microgram of total RNA from each sample was reverse-transcribed into cDNA using an Evo M-MLV RT Kit with gDNA Clean for qPCR (Accurate Biology, Hunan, China), following the manufacturer’s protocol. Each qRT-PCR reaction mixture contained 5 μL of 2× SYBR Green, 4.5 μL of RNase-free water, 0.1 μL of diluted cDNA, and 0.2 μL each of forward and reverse primers (10 μM). The 16S rRNA gene served as the endogenous control. Relative expression levels were calculated using the 2^−ΔΔCT^ method, with comparisons made against the control group. A complete list of the primers used in this work is provided in Table S3.

### 2.16. Measurement of GFP Fluorescence Intensity

The measurement of promoter activity via GFP intesntity was performed as previously described [23]. Single colony of wild-type *E*. *tarda* EIB202 (*n* = 6) and the *ΔpckA* mutant (*n* = 6) harboring the reporter plasmids (pMW82-*esrC*p or pMW82-*evpA*p) were individually inoculated into 1 mL of Tryptic Soy Broth (TSB) supplemented with 100 μg/mL ampicillin. For the wild-type cultures, the clones were divided into two groups: an untreated control group (*n* = 6) and a treatment group supplemented with 25 mM OAA (*n* = 6). The bacterial cultures were incubated overnight at 30 °C with shaking until saturation, followed by static incubation at room temperature for 24 h. For flow cytometry analysis, 1 μL of each bacterial culture was diluted into 1 mL of sterile physiological saline (0.89% NaCl). The mean fluorescence intensity (MFI) of GFP at the single-cell level was quantified using a CytoFLEX flow cytometer (Beckman Coulter, Brea, CA, USA). The GFP signal was collected in the FITC channel (excitation 488 nm, emission 530/30 nm), and the mean FITC-A value of the target bacterial population (gated as P2) was recorded for subsequent analysis. A minimum of 10,000 events were recorded for each sample.

### 2.17. Determination of Relative Plasmid Copy Number (PCN) and Fluorescence Normalization

To eliminate the confounding effect of altered plasmid maintenance caused by metabolic burden in the *ΔpckA* mutant, the raw GFP fluorescence intensity was normalized to the relative PCN. Total genomic DNA, including episomal plasmids, was extracted using a bacterial genomic DNA extraction kit (TIANGEN Biotech, Beijing, China). Quantitative real-time PCR (qPCR) was performed using specific primers targeting the plasmid-encoded *gfp* gene (reporter gene) and the chromosome-encoded gyrB gene (single-copy reference). The amplification efficiencies of the primers were evaluated via standard curves. The relative PCN of the mutant strain compared to the wild-type strain was calculated using the Pfaffl mathematical model [46]. Finally, the intrinsic promoter activity was determined by dividing the raw mean fluorescence intensity (MFI) by the relative PCN [47].

### 2.18. The Effect of Vaccine Monitoring and Calculations

The protective efficacy of the *ΔpckA* mutant was evaluated using zebrafish (*n* = 18) and tilapia (*n* = 12) models as previously described [48]. Bacteria were subcultured in TSB medium until reaching an OD_600_ of 0.7, and the precipitate was collected and resuspended in 0.85% sterile saline to an OD_600_ = 1.0. For the immunization group, zebrafish and tilapia were intraperitoneally injected with the *ΔpckA* mutant at doses of 1.4 × 10^5^ and 1.4 × 10^6^ CFU/fish, respectively. Corresponding control groups were injected with an equal volume of sterile PBS. Following the immunization period (7 days for zebrafish and 14 days for tilapia), both the immunized and control fish were challenged with wild-type *E. tarda* EIB202 via *i.p*. injection. The challenge doses were 1.2 × 10^5^ CFU/fish for zebrafish and 1.9 × 10^6^ CFU/fish for tilapia. Mortality was monitored daily for 20 days post-challenge. The relative percent survival (RPS) was calculated using the following formula:

RPS = [1 − (percent mortality of immunized fish/percent mortality of control fish)] × 100

### 2.19. Statistical Analysis

Statistical analysis was conducted using IBM SPSS Statistics version 26.0 and GraphPad Prism 8.0. All data are shown as the mean ± SD. Every experiment was repeated at least three times independently. Differences between two groups were assessed with an unpaired Student’s *t*-test. Thresholds for statistical significance were established at *p* < 0.05 and *p* < 0.01. Graphs were produced with GraphPad Prism 8.0.

## 3. Results

### 3.1. Phosphoenolpyruvate Carboxykinase (PEPCK) Mediates Bacterial Virulence

Our previous studies have demonstrated the role of the pyruvate cycle in mediating antibiotic resistance [30]; however, other functions of this cycle remain poorly understood. PEPCK is situated at a key node that distinguishes the pyruvate cycle from the TCA cycle. Therefore, we generated a deletion mutant, *ΔpckA*, in *E. tarda* EIB202. We first assessed the antibiotic susceptibility of the *ΔpckA* mutant to a panel of antibiotics using MIC assays. The mutant exhibited an altered antibiotic susceptibility profile compared with the wild-type EIB202; specifically, it showed decreased susceptibility to clindamycin and sulfamethazine (as indicated by elevated MIC values), while demonstrating increased susceptibility to the remaining tested antibiotics, confirming the role of PEPCK in modulating antibiotic susceptibility in a compound-dependent manner (Figure S1).

**Figure 1:**
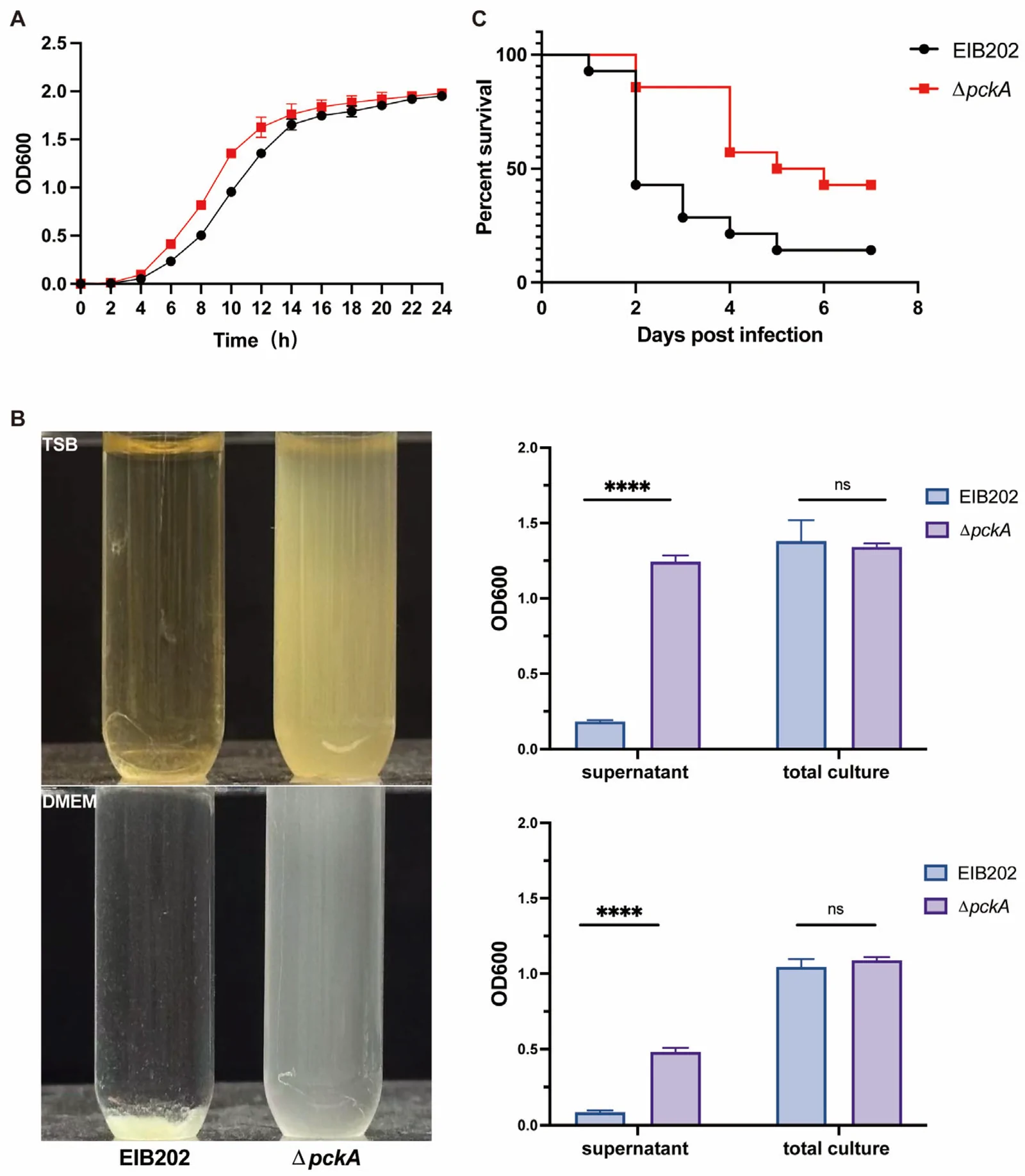
Distinct growth dynamics and virulence-associated phenotypes between *E. tarda* EIB202 and the *ΔpckA* mutant. (**A**) Growth curves of EIB202 and *ΔpckA*. (**B**) Auto-aggregation of EIB202 and *ΔpckA* cultured in TSB and DMEM. Aggregation was assessed at 24 h post-subculture by measuring the OD_600_ measurement after thorough resuspension of the entire culture. (**C**) Survival rates of tilapia injected with 2 × 10^6^ CFU of EIB202 or *ΔpckA*. Data are presented as the mean ± SD from at least three independent experiments. Statistical significance was determined using an unpaired Student’s *t*-test (**** *p* < 0.0001; ns, not significant).

During growth curve experiments, we observed that *ΔpckA* grew faster than EIB202 during the exponential phase. For example, at 10 h post-inoculation, the OD_600_ reached 1.0 for EIB202 but 1.3 for *ΔpckA*. Additionally, *ΔpckA* entered stationary phase approximately 4–6 h earlier than EIB202 (Figure 1A), suggesting that PEPCK deletion perturbs the physiological state of the bacterium. Interestingly, EIB202 exhibits auto-aggregation, a typical virulence feature of *E. tarda*, whereas *ΔpckA* displayed a dispersed phenotype and failed to form auto-aggregates (Figure 1B). But their OD_600_ were similar if mixed thoroughly (Figure 1B). Similar experiments performed in DMEM medium yielded the same results as those in TSB medium, further suggesting reduced or lost virulence in the mutant (Figure 1B).

To confirm the loss of virulence, Nile tilapia were challenged with equal doses of EIB202 and *ΔpckA*. Consistent with the aggregation results, EIB202-infected fish had a survival rate of 10%, while those infected with *ΔpckA* showed a survival rate of 50% (Figure 1C). These data indicate that PEPCK not only regulates aminoglycoside sensitivity but also influences bacterial virulence.

### 3.2. Proteomic Analysis of ΔpckA

**Figure 2:**
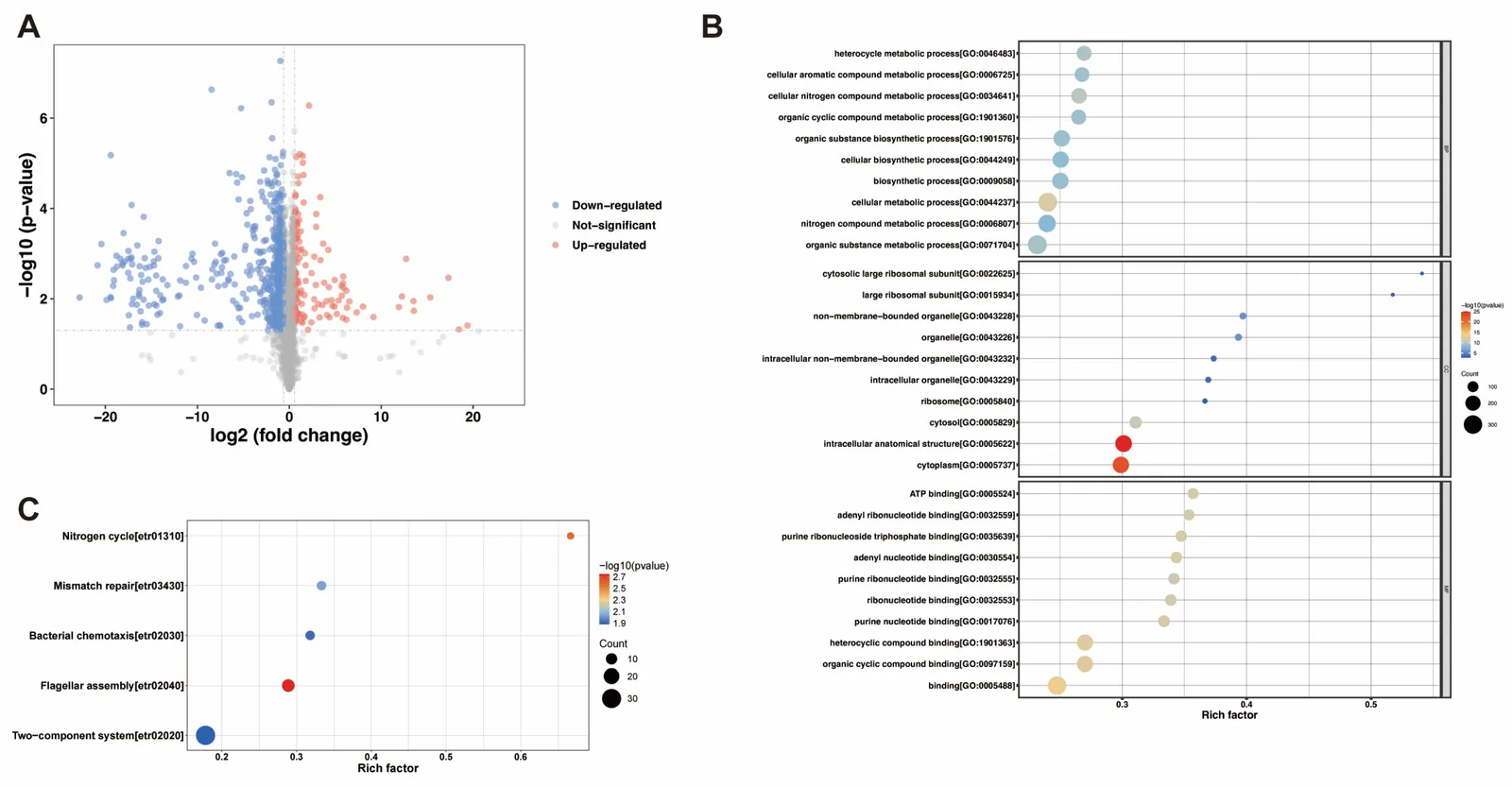
Proteomic analysis of *E. tarda* EIB202 and the *ΔpckA* mutant. (**A**) Volcano plots showing differential protein expression between EIB202 and *ΔpckA*. Downregulated proteins are shown in blue, and upregulated proteins in red (|log_2_FC| ≥ 1.5, *p* < 0.05). (**B**) Bioinformatics analysis of DEPs in *ΔpckA*, including GO annotations for CC, MF and BP. (**C**) KEGG enrichment analysis of DEPs between EIB202 and *ΔpckA*.

To investigate the mechanism underlying *ΔpckA*-mediated virulence, we performed label-free quantitative proteomic analysis on EIB202 and *ΔpckA* cultured in growth medium until the OD_600_ reached 1.0. Only proteins with at least two matched peptides and a false discovery rate ≤ 0.01 were considered identified. A total of 2716 proteins were identified in each sample. Differentially expressed proteins (DEPs) were defined as up-regulated if the fold change (FC) in *ΔpckA* was ≥1.50, or down-regulated if the FC was ≤0.67 (Figure 2A). Using these criteria, 1291 DEPs were identified, including 386 up-regulated and 905 down-regulated proteins (Table S1). These DEPs accounted for 28% of all identified proteins, indicating a global shift upon PEPCK deletion.

We next performed Gene Ontology (GO) analysis on the DEPs to explore functional categories, including biological processes (BP), cellular components (CC), and molecular functions (MF). In the BP category, ten processes were enriched, including organic substance metabolic process, cellular metabolic process, heterocycle metabolic process, organic cyclic compound metabolic process, cellular aromatic compound metabolic process, response to stimulus (cellular response also listed), as well as bacteria-type flagellum-dependent cellular motility, cilium- or flagellum-dependent motility, and archaeal or bacterial-type flagellum-dependent motility. Notably, in the CC enrichment analysis, five components were associated with the bacterial flagellum or cell projections. Combined with the MF enrichment for ATP binding and purine nucleotide binding, these results suggest that *ΔpckA* affects the cellular motility apparatus, which is highly energy-consuming (Figure 2B). Furthermore, Kyoto Encyclopedia of Genes and Genomes (KEGG) pathway analysis revealed five enriched pathways (Figure 2C). Of particular interest were flagellar assembly and bacterial chemotaxis, which were consistent with the GO analysis. Taken together, these results demonstrate alterations in the flagellar system and two-component systems upon PEPCK depletion.

### 3.3. Protein-Protein Interaction (PPI) Analysis

**Figure 3:**
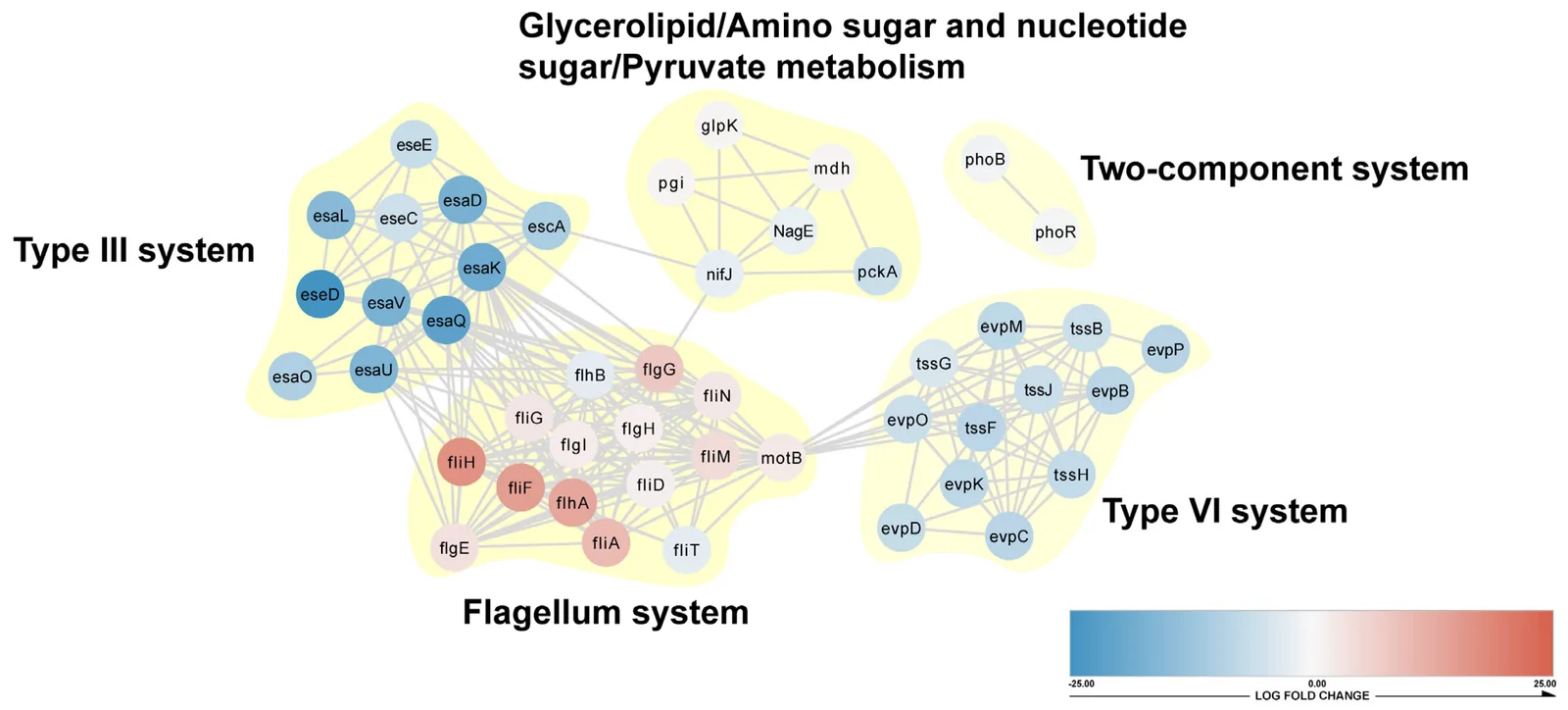
Protein–protein interaction (PPI) network of differentially expressed proteins. Interactions were analyzed using the STRING database. Gray shaded regions indicate proteins enriched in KEGG pathways. Downregulated and upregulated proteins are represented by blue and red circles, respectively.

To gain insight into how the altered proteins are interconnected, particularly those related to the secretion system, we performed protein–protein interaction PPI analysis. Five distinct PPI networks were identified (Figure 3). Interestingly, in addition to pathways involved in glycerolipid metabolism, amino sugar and nucleotide sugar metabolism, pyruvate metabolism, and the two-component system, the flagellar system was enriched, along with two other secretion systems—type III secretion system (T3SS) and type VI secretion system (T6SS). Expression levels of T3SS and T6SS were decreased in *ΔpckA*, whereas flagellar system components showed increased expression. Given that T3SS and T6SS are critical for *E. tarda* virulence, the reduced expression of these two secretion systems likely explains the attenuated virulence observed in *ΔpckA*. Thus, our proteomic analysis suggests that PEPCK may regulate *E. tarda* virulence through modulation of both secretion systems and the flagellar system.

### 3.4. ΔpckA Exhibits Increased Cellular Motility

**Figure 4:**
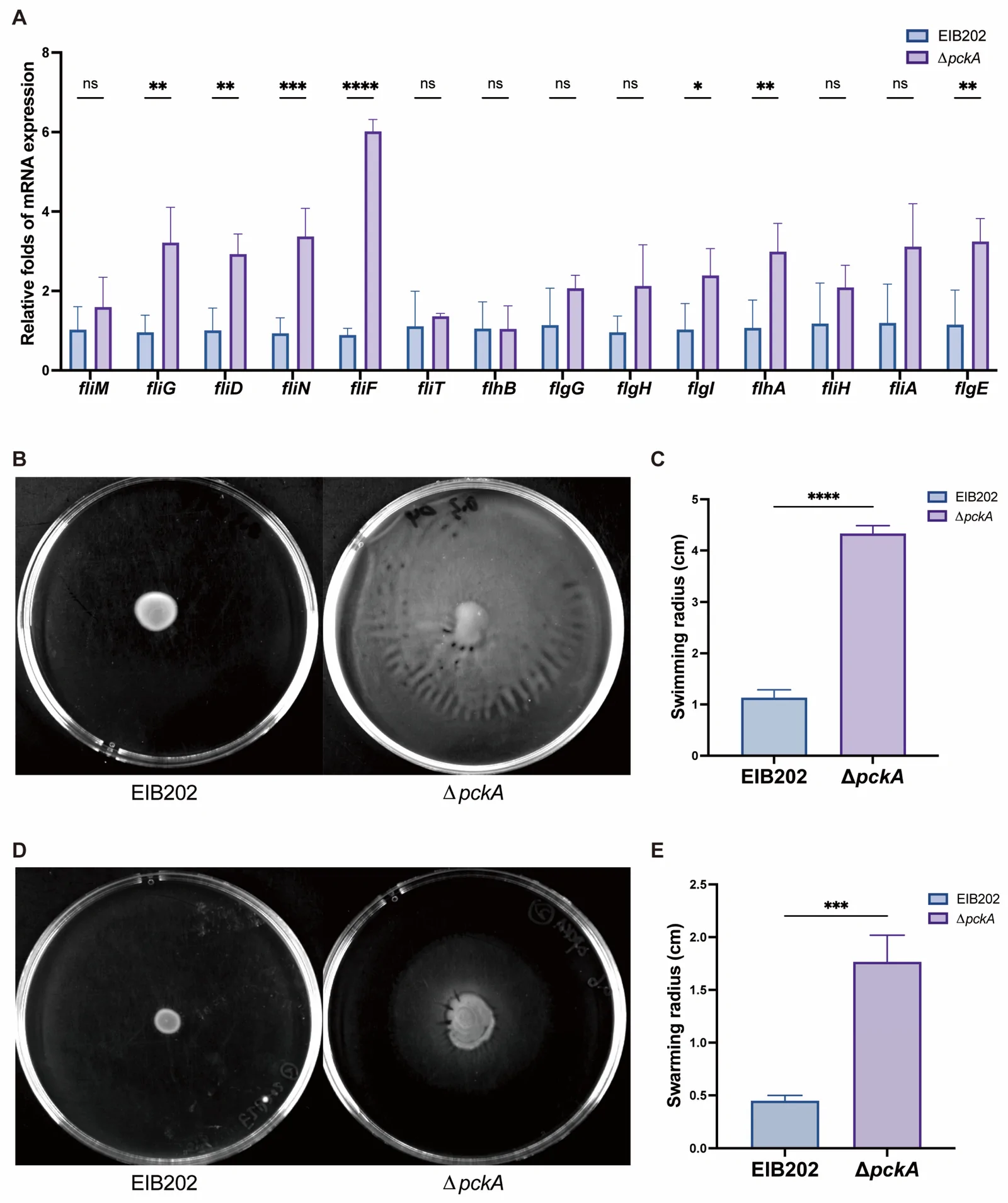
Motility of *E. tarda* EIB202 and the *ΔpckA* mutant. (**A**) mRNA levels of flagellar genes in EIB202 and *ΔpckA* examined by qRT-PCR. (**B**) Photographs of the swimming assay. (**C**) Histograms of ureswimming assay radii. (**D**) Photographs of the swarming assay. (**E**) Histograms of swarming assay radii. Data are presented as mean ± SD from at least three independent experiments. Statistical significance was determined using multiple unpaired *t*-tests with Holm–Šidák correction (**A**) or unpaired Student’s *t*-test (**C**,**E**) (* *p* < 0.05, ** *p* < 0.01, *** *p* < 0.001, **** *p* < 0.0001; ns, not significant).

Flagella provide motility, enabling bacteria to navigate toward their preferred niche within the host. This capability is typically required during the initial stages of infection but is often downregulated upon cellular invasion. To investigate the role of PEPCK in regulating bacterial motility, we examined the expression of flagellar genes. The transcript levels of *fliG*, *fliD*, *fliN*, *fliF*, *flgL*, *flhA*, and *flgE* were all elevated in *ΔpckA* compared to the wild-type strain (Figure 4A). To further confirm the motility phenotype, we analyzed the swimming and swarming behaviors of both strains. Compared with EIB202, *ΔpckA* was highly motile, spreading across the plate after 16 h of incubation and showing approximately a fourfold increase in swimming motility (Figure 4B). Similarly, in swarming assays, *ΔpckA* exhibited greater swarming ability than EIB202 (Figure 4C). Altogether, these data indicate that the motility of EIB202 is lower than that of *ΔpckA*.

### 3.5. T3SS and T6SS Were Decreased in ΔpckA

**Figure 5:**
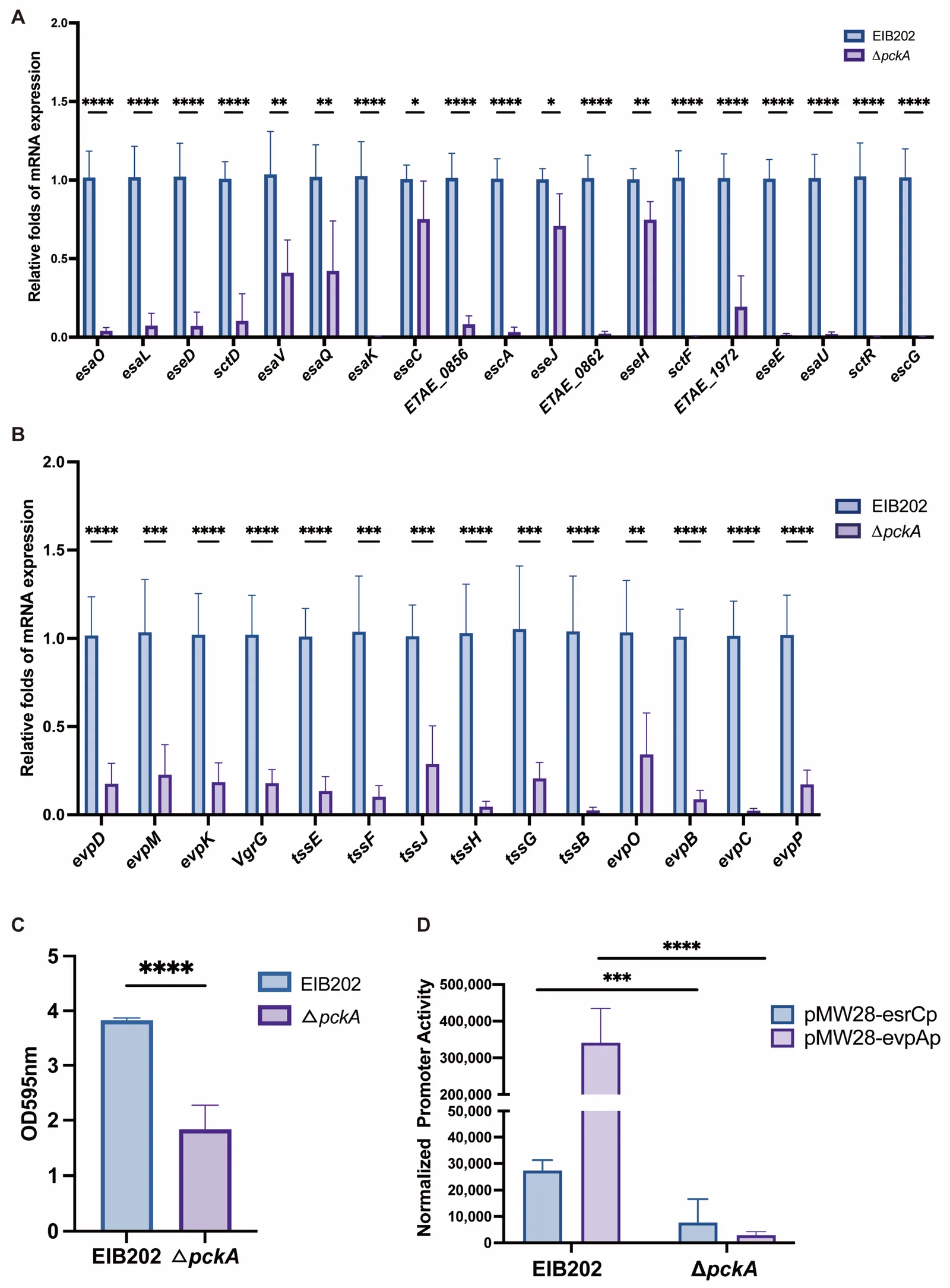
Deletion of *pckA* attenuates the pathogenic traits of *E. tarda* EIB202. (**A**) mRNA levels of T3SS genes in EIB202 and *ΔpckA* quantified by qRT-PCR. (**B**) mRNA levels of T6SS genes in EIB202 and *ΔpckA* quantified by qRT-PCR. (**C**) Biofilm formation assessed by crystal violet staining. Strains were subcultured in DMEM in a 24-well plate containing 12 mm coverslips. At 24 h post-subculture, bacteria adherent to coverslips were fixed and stained with 0.1% crystal violet. The stained dye was dissolved and quantified by optical density measurement. (**D**) Promoter activities of *esrC* and *evpA*. Raw GFP fluorescence was normalized to relative plasmid copy number (PCN). Data are presented as mean ± SD from at least three independent experiments. Statistical significance was determined by multiple unpaired *t*-tests with Holm-Sidak correction (**A**,**B**) or an unpaired Student’s *t*-test (**C**,**D**) * *p* <0.05, ** *p* <0.01, *** *p* <0.001, **** *p* <0.0001).

To examine the transcriptional levels of T3SS and T6SS, we performed qRT-PCR to measure gene expression in EIB202 and *ΔpckA*. For the entire T3SS gene cluster, the expression of *esaO*, *esaL*, *eseD*, *sctD*, *esaK*, *ETAE_0856*, *escA*, *sctF*, *ETAE_1972*, *eseE*, *esaU*, *sctR*, and *escG* was decreased by at least two-fold in *ΔpckA*, while the expression of *esaV*, *esaQ*, *eseC*, *eseJ*, and *eseH* decreased by 1.34–1.42-fold (Figure 5A). Similarly, expression of T6SS genes, including *evpD*, *evpM*, *evpK*, *vgrG*, *tssE*, *tssF*, *tssJ*, *tssH*, *tssG*, *tssB*, *evpO*, *evpB*, *evpC*, and *evpP*, was reduced by at least two-fold in *ΔpckA* (Figure 5B). T3SS and T6SS are also associated with biofilm formation in *E. tarda*. Consistently, biofilm formation was decreased upon PEPCK deletion (Figure 5C).

Combining the proteomic and transcriptional analyses, we observed that the entire T3SS and T6SS gene clusters were downregulated, suggesting that PEPCK may affect the transcriptional regulators of these two clusters. In *E. tarda*, T3SS and T6SS are regulated via the EsrA-EsrB two-component system and its downstream regulator, EsrC. To test this possibility, we constructed reporter plasmids using the promoter-less vector pMW82, which carries an unstable GFP to assess promoter activity. Specifically, we generated reporters for the *esrC* promoter (representing T3SS regulation) and the *evpA* promoter (representing T6SS). To eliminate potential artifacts arising from differences in plasmid maintenance between strains, the raw mean fluorescence intensity (MFI) was normalized to the relative plasmid copy number (PCN; Figure S2). Consistent with the transcriptional expression data, GFP fluorescence intensity was decreased in *ΔpckA* (Figure 5D). These results indicate that T3SS and T6SS are downregulated in *ΔpckA*.

### 3.6. Oxaloacetate Negatively Regulate Bacterial Virulence

**Figure 6:**
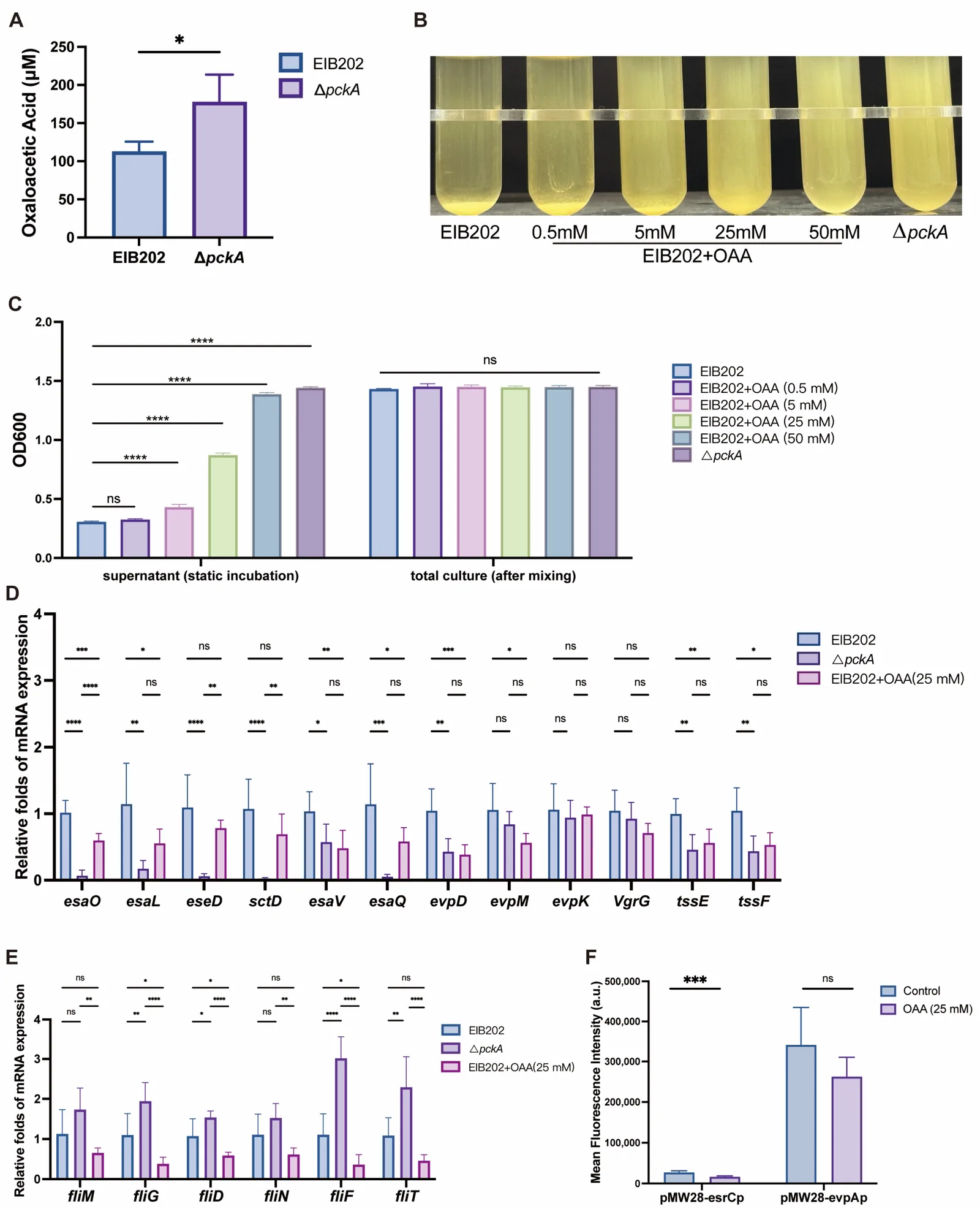
Oxaloacetate (OAA) negatively regulates bacterial virulence. (**A**) The OAA content in EIB202 and *ΔpckA*. (**B**) Exogenous OAA inhibits auto-aggregation of *E. tarda* EIB202 in a dose-dependent manner. (**C**) OD_600_ measurements confirm that OAA suppresses auto-aggregation (resulting in a more turbid static supernatant) without affecting overall bacterial growth. (**D**) OAA treatment significantly downregulates transcriptional expression of virulence-related T3SS and T6SS genes. (**E**) In contrast to its effect on secretion system genes, exogenous OAA has minimal impact on flagellar gene expression. (**F**) OAA reduces the transcriptional activity of *esrC* and *evpA* promoters. Data are presented as mean ± SD from at least three independent experiments. Statistical significance was determined using an unpaired Student’s *t*-test (**A**,**C**,**F**) or two-way ANOVA with Tukey’s multiple comparisons test (**D**,**E**). For panel C, all experimental groups were compared to the EIB202 control group. (* *p* < 0.05, ** *p* < 0.01, *** *p* < 0.001, **** *p* < 0.0001; ns, not significant).

PEPCK converts oxaloacetate (OAA) to phosphoenolpyruvate (PEP); consequently, the *ΔpckA* mutant accumulates high levels of OAA (Figure 6A). To investigate whether OAA influences virulence, we first examined the auto-aggregation phenotype. OAA treatment reduced *E. tarda* auto-aggregation in a dose-dependent manner, as indicated by increased culture turbidity (Figure 6B and Figure S3A). The OD_600_ of the culture supernatant (after overnight settling) showed an OAA-dependent increase, whereas the OD_600_ remained unchanged when the cultures were thoroughly mixed (Figure 6C).

To rule out the possibility that OAA alters medium viscosity and physically hinders bacterial sedimentation, we performed a control experiment using 0.1% glycerol, which exhibited a viscosity comparable to that of 25 mM OAA as determined by a soft centrifugation method (Figure S3B). Supplementation with 0.1% glycerol partially increased supernatant turbidity (OD_600_ = 0.534 ± 0.033), whereas OAA-treated cultures remained significantly more turbid (OD_600_ = 0.872 ± 0.017) compared to the untreated EIB202 control (OD_600_ = 0.307 ± 0.006) (Figure S3C). Notably, the presence of 0.1% glycerol did not affect the turbidity of the non-aggregating *ΔpckA* mutant (Figure S3C), indicating that glycerol itself does not interfere with bacterial physiology or sedimentation. These results indicate that while the viscosity of OAA might partially contribute to the increased supernatant turbidity, the effect of OAA on autoaggregation is predominantly mediated through metabolic regulation rather than physical interference.

Furthermore, OAA treatment decreased the transcriptional expression of T3SS and T6SS genes (Figure 6D), but had only a minor effect on flagellar gene expression (Figure 6E). Consistent with the gene expression data, OAA reduced the transcriptional activity of the pMW82-*esrC*p and pMW-*evpA*p reporters (Figure 6F). However, OAA did not affect cellular motility. Together, these data suggest that the accumulation of OAA in the *ΔpckA* mutant suppresses T3SS and T6SS expression without influencing the flagellar system.

### 3.7. ΔpckA Is a Potential Live Vaccine

**Figure 7:**
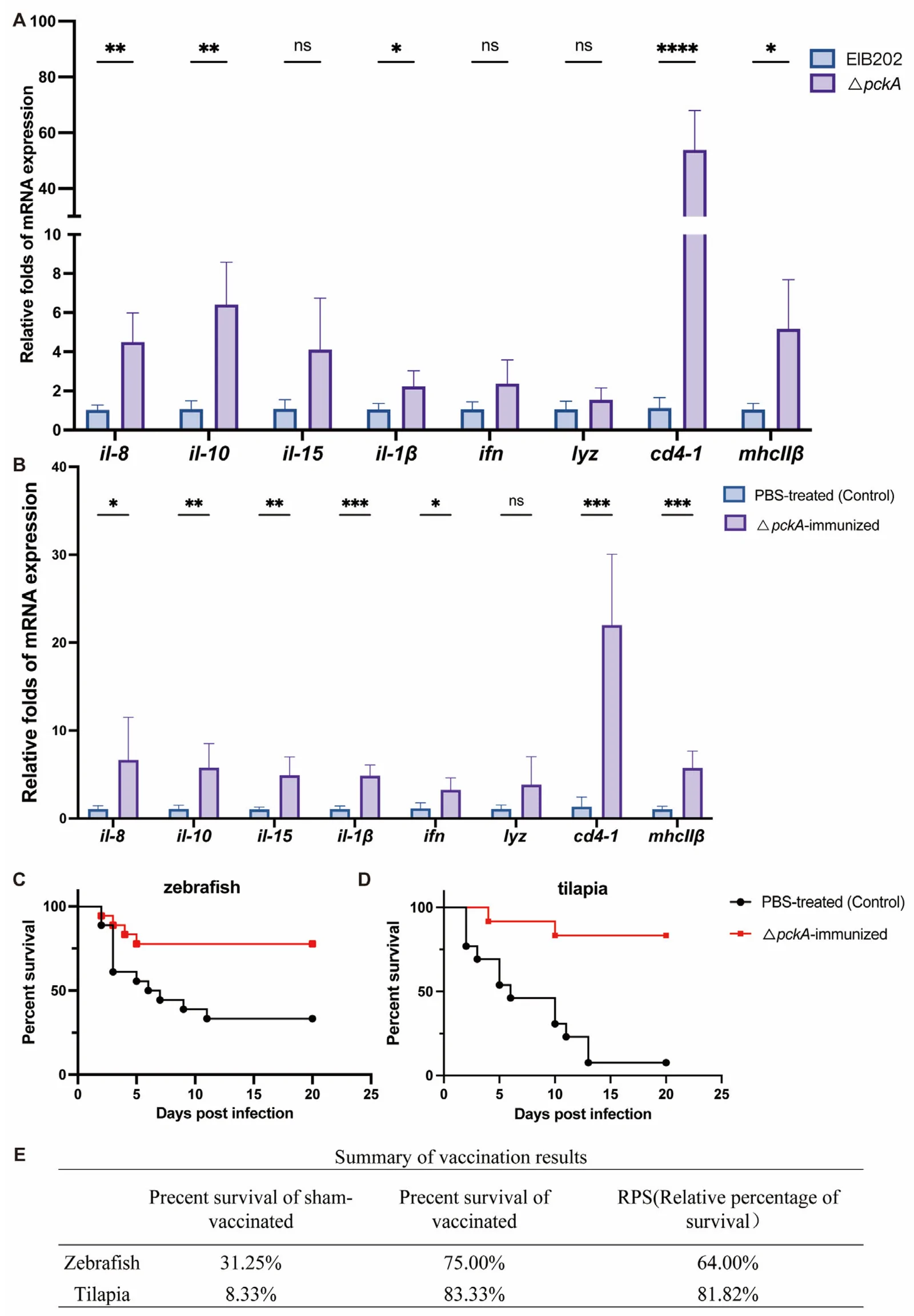
Evaluation of the *ΔpckA* mutant as a potential live attenuated vaccine. (**A**) *ΔpckA* induces a robust early immune response under sublethal infection. (**B**) Immunization with *ΔpckA* significantly upregulates immune gene expression. (**C**) Vaccination with *ΔpckA* improves the survival rate of zebrafish against lethal.challenge. (**D**) Vaccination with *ΔpckA* improves the survival rate of tilapia against lethal challenge. (**E**) Summary of vaccination outcomes in zebrafish. Data are presented as mean ± SD from at least three independent experiments. Statistical significance was determined using multiple unpaired *t*-tests with Holm–Šidák correction (* *p* < 0.05, ** *p* < 0.01, *** *p* < 0.001, **** *p* < 0.0001; ns, not significant).

Given the reduced virulence of *ΔpckA*, we explored its potential as a vaccine candidate against *E. tarda*. To test this possibility, we first assessed the immune response of zebrafish to *E. tarda* infection. Zebrafish were challenged with a sublethal dose of EIB202 or *ΔpckA*, and immune gene expression was measured at 24 h post-infection. Compared with EIB202, *ΔpckA* induced a much stronger immune response, as evidenced by elevated expression of *il-8*, *il-10*, *il-1β*, *cd4-1*, and *mhcIIβ* mRNAs (Figure 7A). Furthermore, zebrafish immunized with *ΔpckA* showed increased expression of *il-8*, *il-10*, *il-15*, *il-1β*, *ifn*, *cd4-1*, and *mhIIβ* mRNAs, indicating enhanced antigen presentation (Figure 7B). Since no mortality was observed in zebrafish under these challenge conditions, these results suggest that *ΔpckA* elicits an immune response sufficiently strong to confer long-lasting, sterilizing immunity yet mild enough to avoid causing disease in vaccinated fish.

For vaccination studies, both zebrafish and tilapia were used as hosts. Fish were immunized with *ΔpckA* at non-lethal doses of 1.4 × 10^5^ CFU or 1.4 × 10^6^ CFU for 7 or 14 days, then challenged with the LD_50_ dose of EIB202. The relative percent survival (RPS) values for zebrafish and tilapia were 64% and 81.82%, respectively (Figure 7C–E), indicating a good protective effect of this live attenuated vaccine. Taken together, these results suggest that *ΔpckA* represents a promising vaccine candidate.

## 4. Discussion

In this study, we demonstrate that PEPCK, encoded by *pckA*, plays a critical role in coordinating antibiotic sensitivity, metabolic homeostasis, and virulence in *E. tarda*. Our findings reveal that the *ΔpckA* mutant exhibits reduced virulence, increased motility, and enhanced immunogenicity, positioning it as a promising live attenuated vaccine candidate.

Bacterial virulence is intimately linked to metabolic state, yet how metabolism precisely regulates virulence factor expression remains incompletely understood. Emerging evidence indicates that metabolic enzymes and intermediates not only provide energy and biosynthetic precursors but also directly participate in virulence regulation [49]. For instance, deletion of the glutamate synthase gene *gltB* in *Staphylococcus aureus* reduces both antibiotic tolerance and virulence, highlighting glutamate metabolism as a key node connecting resistance and pathogenicity [50]. Similarly, in *E. piscicida*, host-derived mannose-6-phosphate directly activates the transcription factor EvrA, which in turn triggers the virulence regulator EsrB [51]. In *Pseudomonas aeruginosa*, glycerol metabolism influences biofilm and virulence via the Entner-Doudoroff pathway, and disruption of this pathway unexpectedly enhances virulence while inducing a stronger host immune response [52]. Altogether, these studies demonstrate that metabolic reprogramming can either restrict or enhance virulence depending on the context. In our study, the *ΔpckA* mutant grew faster than the wild type yet exhibited significantly reduced expression of T3SS and T6SS, suggesting that PEPCK-mediated metabolic regulation of virulence is not simply a consequence of altered growth rate but rather involves specific metabolic signaling. This is analogous to the finding that succinate promotes EHEC virulence through lysine succinylation [53] and aligns with a recent observation that PEPCK succinylation regulates antibiotic resistance in *Vibrio alginolyticus* [54]. Moreover, in *E. tarda* necrotizing soft tissue infection, nutrient acquisition (e.g., iron, zinc, vitamin B6, and polyamines) is critical for in vivo fitness [55], further supporting the concept that metabolic adaptation is central to bacterial pathogenesis.

PEPCK is the first committed enzyme of gluconeogenesis and has been implicated in virulence in several pathogens. In *Mycobacterium bovis* BCG, *pckA* deletion reduces survival in macrophages and attenuates virulence in mice [56]. In *M. tuberculosis*, PEPCK is essential for growth on fatty acids, and *pckA* deletion leads to rapid clearance in a mouse model, making it a potential drug target [57]. In the plant pathogen *Agrobacterium tumefaciens*, *pckA* is induced by acidic pH and is required for tumor formation [58]. Interestingly, *M. tuberculosis* PEPCK itself induces strong cell-mediated immune responses, increasing CD4^+^ T cells and IFN-γ production, suggesting an immunogenic role beyond metabolism [59]. In our study, *ΔpckA* also exhibits attenuated virulence, but unlike mycobacteria, it grows faster in vitro rather than slower, highlighting species-specific differences in PEPCK dependency. Furthermore, lysine succinylation of PEPCK is elevated in florfenicol-resistant *V. alginolyticus* and directly affects resistance [54], indicating that PEPCK serves as a bridge between metabolism and resistance. Structurally, key residues of *E. coli* PEPCK (e.g., D269, H232, K254, R65, R333) are critical for its catalytic activity [60], providing a foundation for future mechanistic studies in *E. tarda*.

PEPCK catalyzes the reversible conversion of OAA to PEP; therefore, OAA accumulates in the *ΔpckA* mutant. Our experiments show that exogenous OAA phenocopies the *ΔpckA* mutant, suppressing T3SS/T6SS expression and autoaggregation without affecting flagellar motility, indicating that OAA itself acts as a metabolic signal that negatively regulates *E. tarda* virulence. Similar observations have been made in other bacteria. In *Pseudomonas putida*, expression of the GraT toxin leads to TCA cycle downregulation and OAA accumulation, accompanied by growth inhibition [61]. In *Salmonella enterica* serovar Typhimurium, mutants unable to convert malate to both OAA and pyruvate are completely avirulent and protective [62]. In *Enterococcus faecalis*, deletion of oxaloacetate decarboxylase attenuates virulence, indicating that downstream metabolism of OAA is essential for pathogenicity [63]. In *Actinobacillus pleuropneumoniae*, a double deletion mutant lacking PEPC and PEPCK shows reduced virulence in a pig infection model despite normal in vitro carbon fixation [64]. Thus, these observations suggest that OAA is not merely a metabolic intermediate but may function as a signaling molecule in both prokaryotes and eukaryotes. Our study establishs a direct link between OAA accumulation and T3SS/T6SS suppression in *E. tarda*, and we show that OAA acts by reducing the promoter activity of the *evpA*.

One seemingly paradoxical observation in this study is that the *ΔpckA* mutant exhibits enhanced flagellar motility yet significantly attenuated virulence. Bacterial motility is generally considered a virulence-associated trait that facilitates host colonization and niche navigation [65]; however, it is important to note that motility and pathogenicity are not invariably coupled. In many pathogenic bacteria, including *E. tarda*, flagellar motility is required during the initial stages of infection, whereas the establishment of systemic infection and intracellular survival relies heavily on the functional expression of secretion systems, particularly T3SS and T6SS, which deliver effector proteins to subvert host immune defenses [32,34]. Our study revealed that while flagellar proteins were upregulated in *ΔpckA*, the entire T3SS and T6SS gene clusters were globally downregulated, indicating the loss of effector delivery capacity far outweighs any potential benefit conferred by increased motility. Furthermore, motility is typically downregulated upon host cell invasion to avoid immune detection [66]; the sustained hyper-motile state of the mutant may therefore reflect a failure to properly transition to the invasive phase, further compromising its pathogenic potential. This observation is consistent with earlier findings in other bacterial species, where metabolic or regulatory perturbations have been shown to result in similar discordant changes in motility and virulence. For example, in *Ralstonia solanacearum*, a bacterial pathogen causing wilt disease on many plant species, the *motN* mutants exhibit hyper-flagellation but virulence is reduced [67]. Similarly, from a clinical epidemiological perspective, a correlation study on bacterial motility and virulence revealed that higher-motility strains were significantly more prevalent in spontaneous bacterial peritonitis but carried fewer virulence genes, whereas lower-motility isolates required more virulence factors to cause extraintestinal infections, suggesting a compensatory trade-off between motility and virulence [68]. Thus, the enhanced motility in *ΔpckA* does not contradict its attenuated virulence; rather, it underscores the predominant role of T3SS/T6SS in *E. tarda* pathogenesis.

Another notable phenotype caused by *pckA* deletion is the loss of autoaggregation, which correlates inversely with enhanced motility. In *E. tarda*, autoaggregation is primarily mediated by EseB, a translocon component of the T3SS that forms filamentous appendages on the bacterial surface to promote cell–cell adhesion and biofilm formation [37]. Our data showed that OAA accumulation in *ΔpckA* globally downregulates T3SS gene expression, including *eseB*, likely accounting for the loss of autoaggregation. Meanwhile, T3SS downregulation appears permissive for enhanced motility. This inverse correlation is consistent with studies in other bacterial systems: in *Aeromonas hydrophila*, expression of the T3SS represses the lateral flagellar system [69]; the Ag43 autoaggregation protein inhibits motility in *Escherichia coli*, and the SadB regulatory system inversely controls biofilm formation and swarming motility in *Pseudomonas aeruginosa*. [70,71]. From an energetic perspective, PEPCK is a key enzyme in gluconeogenesis and the pyruvate/TCA cycle, and its deletion perturb the cellular energy landscape. Since both T3SS assembly and flagellar biogenesis are energetically costly processes, a metabolic shift caused by OAA accumulation or by the altered energy flux downstream of PEPCK deficiency could redirect resources away from T3SS/aggregation toward flagellar biosynthesis, favoring a motile and dispersal-oriented lifestyle. However, direct experimental evidence linking PEPCK-dependent metabolic changes to energy investment priorities requires further investigation.

An ideal live attenuated vaccine should be avirulent yet immunogenic. Our results show that *ΔpckA* induces a stronger immune response in zebrafish than wild-type EIB202, as evidenced by upregulation of *il-8*, *il-1β*, *ifn*, and *lyz*. This phenomenon that attenuation coupled with enhanced immunogenicity is common among many bacterial vaccine strains. For example, an O-antigen-deficient *Salmonella Typhimurium* attenuated strain induces higher IL-2, IL-10, IL-4, and IFN-γ expression in chicken macrophages and provides effective protection [72]. *Listeria monocytogenes actA* and *plcB* double deletion mutants are highly attenuated yet efficiently induce CD8^+^ T cell memory and long-lasting protection [73]. In *Francisella tularensis*, the Δ*clpB* mutant induces seroconversion and pulmonary IL-17 levels that correlate with protection [74]. In *Vibrio alginolyticus*, a *tyeA*, the T3SS regulator, deletion mutant shows 40-fold reduced virulence and upregulates IgM, IL-1β, IL-6, and TNF-α in zebrafish, providing 71.2% relative protection [75]. Similarly, a peptide aptamer targeting SmpB in *Aeromonas veronii* induces strong antibody responses and protects zebrafish from lethal challenge [76]. These studies indicate that rational deletion of metabolic or virulence genes often exposes immunogenic antigens or generates enhanced inflammatory signals, leading to superior immune responses compared to wild-type strains. The strong immunogenicity of *ΔpckA* in our study may be attributed to its increased flagellar motility and metabolic reprogramming, offering a new strategy for developing metabolically attenuated live vaccines.

## 5. Conclusions

In summary, we identify PEPCK as a key metabolic regulator of virulence in *E. tarda*, acting through OAA accumulation to suppress T3SS/T6SS expression while enhancing motility and immunogenicity. The *ΔpckA* mutant exhibits a favorable safety and protection profile, making it a promising live attenuated vaccine candidate for aquaculture. Future work should explore whether OAA directly binds to EsrC/EvpA or other regulatory proteins, and whether this regulatory mechanism is conserved in other bacterial pathogens.

## Data Availability

The data supporting the findings of this study are available from the authors upon reasonable request. The proteomic data was deposited in the ProteomeXchange Consortium (https://proteomecentral.proteomexchange.org, accessed on 6 May 2026) via the iProX partner repository with the accession number of PXD078087. The data that supports the findings of this study are available in the Supporting Information of this article.

## Supplementary Materials

The following supporting information can be downloaded at: https://www.mdpi.com/article/10.3390/biology15161338/s1, Figure S1: Deletion of *pckA* alters the antibiotic susceptibility profile of *Edwardsiella tarda* EIB202; Figure S2: Relative plasmid copy number (PCN) in the *ΔpckA* mutant; Figure S3: Exogenous OAA inhibits *E. tarda* auto-aggregation via metabolic regulation rather than physical interference, without altering overall bacterial growth; Table S1: List of differentially expressed proteins; Table S2: Gene knockout primers used in this study; Table S3: qRT-PCR primers used in this study.
