# Supplementary material for: Phosphoenolpyruvate Carboxykinase Controls Edwardsiella tarda Virulence via Oxaloacetate
Source: Biology (Basel). 2026 Aug 7;15(16):1338. doi: 10.3390/biology15161338 (PMC13509319; doi:10.3390/biology15161338)
Supplement: Supplementary file 1 [file biology-15-01338-s001.zip › Supplementary material.pdf]

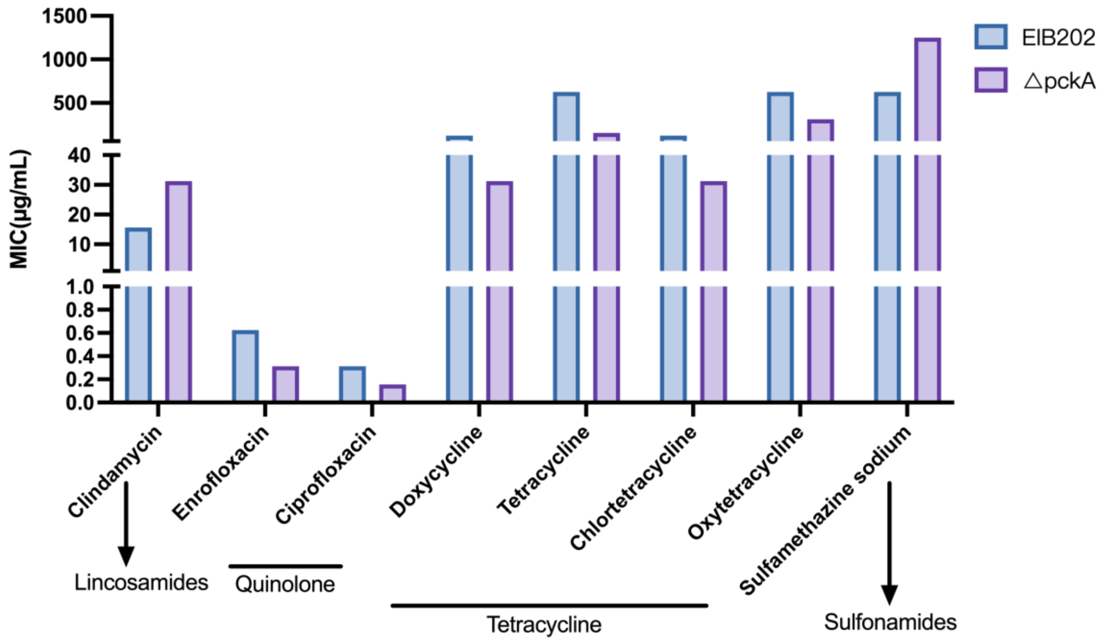

Figure S1 Deletion of *pckA* alters the antibiotic susceptibility profile of *Edwardsiella tarda* EIB202. The minimum inhibitory concentrations (MICs) of eight different antibiotics, categorized into four major classes (lincosamides, quinolones, tetracyclines, and sulfonamides), were determined for the wild-type EIB202 and the *ΔpckA* mutant. The y-axis represents the MIC values (μg/mL) and is broken to accommodate the wide range of concentrations.

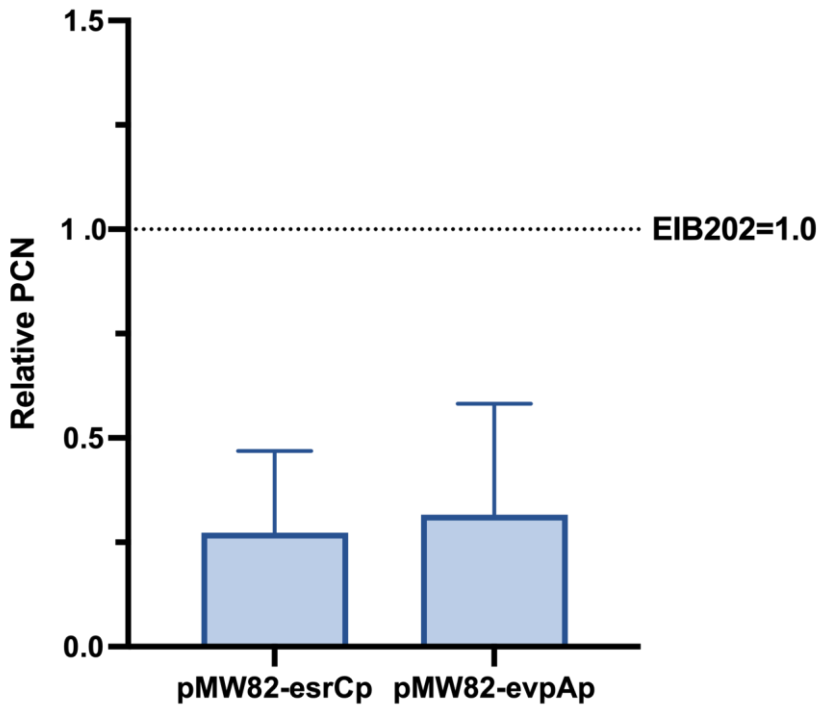

Figure S2 Relative plasmid copy number (PCN) in the *ΔpckA* mutant. The relative PCN of the reporter plasmids (pMW82-esrCp and pMW82-evpAp) in the *ΔpckA* mutant was quantified by real-time qPCR. The horizontal dotted line at Y = 1.0 represents the normalized baseline of the wild-type EIB202 strain. Data are presented as the mean ± standard deviation (SD) from independent biological replicates (n = 6).

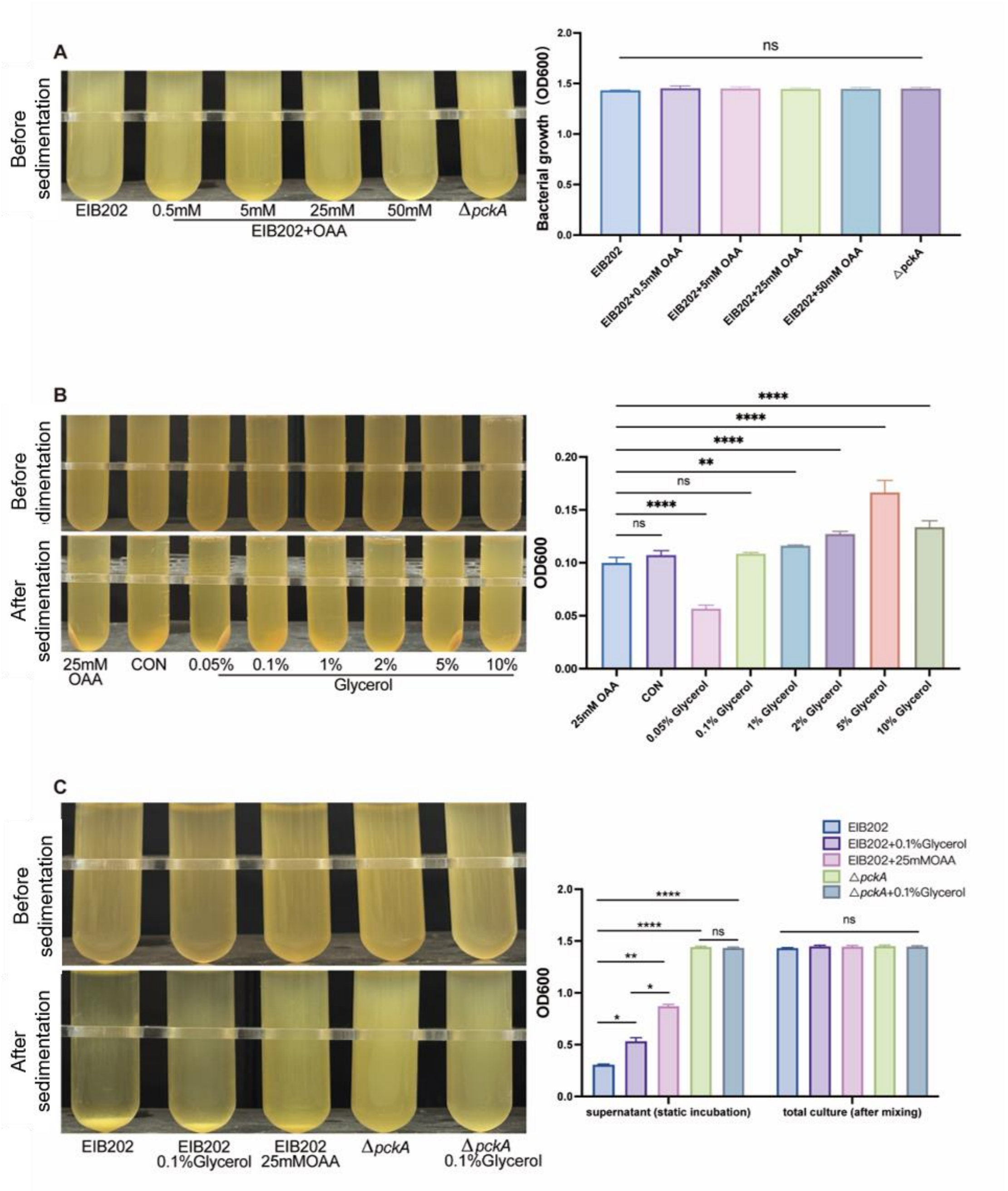

Figure S3 Exogenous OAA inhibits *E. tarda* auto-aggregation via metabolic regulation rather than physical interference, without altering overall bacterial growth. (A) Supplementation of exogenous OAA does not affect the overall growth of EIB202. Representative images before static incubation and total culture OD600 measurements show no significant differences across treatments. (B) Photographs of cultures before and after 500 × g centrifugation, alongside supernatant and total OD600, were recorded for E. tarda treated with 25 mM OAA or 0-10% glycerol to screen for the optimal

physical viscosity control. (C) Representative images before and after incubation in the presence of OAA or 0.1% glycerol, and the corresponding OD600 of the supernatant and total culture of EIB202 and Δ*pckA* strains. Data are presented as the mean ± SD from at least three independent experiments. Statistical significance was determined using ordinary one-way ANOVA. For post-hoc analysis, Dunnett's multiple comparisons test was used for (A) and (B), with the untreated EIB202 group serving as the reference control in (A) and the 25 mM OAA group in (B). For (C), Sidak's multiple comparisons test was used for preselected pairwise comparisons. (*$P < 0.05$, **$P < 0.01$, ***$P < 0.001$, ****$P < 0.0001$; ns, not significant).

Table S2 Gene knockout primers used in this study

Primer name	Primer sequences(5' → 3')
pckA-F	CCACATACAGTAAACGCAGATAAAAATAACCTACAAGGAGT AGTTCAATGATTCCGGGGATCCGTCGACC
pckA-R	CGTTGAGCGGCCGATATCCGCGTGGGCGTGCGTTAGCGCTG AGGGCCGGCTGTAGGCTGGAGCTGCTTCG
pckA-KO-F	GATTATCAAAATGGAAGGATCTGCCACAAAA
pckA-KO-R	TGAAGATCCGAGCTTATTTACGACGG

Table S3 qRT-PCR primers used in this study

Genes	Primer sequences(5' → 3')
esaO-F	ATGGAGAGCGAATCACAGCG
esaO-R	GCTCTCCTCCAGCAGTTTCA
esaL-F	GCGCCATGCTACACCTCTTA
esaL-R	GCAATAATCCTCCAGGCCGA
eseD-F	GGCGCATACCATTAGCAACG
eseD-R	CGCCTTTTCCAGCGTTTCAT
sctD-F	TTTATCGGTATGGCGGGAGC
sctD-R	TGGCCGTAATCTGGACGATG
esaV-F	CAGCAGGAGAATCAGGCGAT
esaV-R	AGCTCCTGAAACGAGAGCAC
esaQ-F	CATACGCTGCCGTTAGTCCT
esaQ-R	TATCGGGAGAGGAGAGCCAG
esaK-F	CCCAGCAAAAGCAGGAGGAG
esaK-R	CAGCGCATTTTGGTCCTGTT
eseC-F	TTCTGGACATCTTTGGCCCC
eseC-R	TTTGAGATTGGCGTTGTCGC
ETAE_0856-F	CAGGCGATCAAGGGGGAAAT
ETAE_0856-R	ATCTGTGGGTGATCGTGCTG
escA-F	GATCAGGCCGTCTTCTGCTT
escA-R	CCCTTGATCGCCTGGTACTG
eseJ-F	GCGAGGATACAGATGGCGAA
eseJ-R	TCAAACTGCTCACCGACCTC
ETAE_0862-F	GCCTGATCGACTGCCTGAAT
ETAE_0862-R	CAGACACGACTGACGCTGAC
eseH-F	CAAGTTCCACATCAGCGTGC
eseH-R	CATACAGCGTAAACTGCGCC
sctF-F	GTCTCTCAGCTGGGCAATCA
sctF-R	GCAAACTGGGCTTTCAGCAT

ETAE_1972-F	GCGGCTTCGGCATTAACTAC
ETAE_1972-R	ATGTCCAGCACCTCCATGAC
eseE-F	CCTCTGTGACTTTACCGCCC
eseE-R	CGTCGTCGCATCCTATCCAG
esaU-F	AGACCGGCTTTTTGTTTGCG
esaU-R	CAGGCCGAGGATCACGATTT
sctR-F	CTGGTGGAGGCGTTTCAGAT
sctR-R	GAACGGCATGGAGATGGTCA
escG-F	TGCGAAGCCATTCTGCTCTT
escG-R	CGCGTTTTCTCCTGAGTGGT
evpD-F	CCCAGATGAAAGGAACCCCC
evpD-R	ACCGCCAGCGTTTTCAGATA
evpM-F	GGCTGAAAAAGCTGGACACG
evpM-R	TTTCAACCTCGCTCTCCCAC
evpK-F	CAACTGGGTCGAGGTGATCC
evpK-R	TACATCTCCGCCCAATAGCG
VgrG-F	GCCGACTGATGGAGGAAGAG
VgrG-R	TATCATCGCCGGATTCGCTC
tssE-F	GATCTGTTTGAACCCCGGCT
tssE-R	GAAACATCCAGCACGGCATC
tssF-F	GGCCAAACTGACCGATACCA
tssF-R	CTGACGGCATCGACCGAATA
tssJ-F	CAGGCCATCTTGGTGAATGC
tssJ-R	CTGCAAAATTCCGTCAGGGG
tssH-F	CCTGTCGGAGTTTCAGGAGG
tssH-R	GGGCTTTCTCCACCTCATCC
tssG-F	TCGAGATCGCCGTCACATTC
tssG-R	ATACCACTTCGTCGGCATCC
tssB-F	TACCCTGAGCGGAGATGACA

tssB-R	CAATACCTCACCCAGGCGTT
evpO-F	GGTATCTCGGCGTATCGGTC
evpO-R	TGCGCGTTTCTGTTTTCCAG
evpB-F	TATCGCCCTGACCCACTGTA
evpB-R	GATTTTGTCGCGGACCATCG
evpC-F	GAAGTGCTGGGTTTTGCCTG
evpC-R	CTCCAGCGTGGGAAAGTTCT
evpP-F	CCCAGATGAAAGGAACCCCC
evpP-R	ACCGCCAGCGTTTTCAGATA
fliM-F	CTACGATCCCACCACCCAAC
fliM-R	ACGGGCAAACTCATGGTAGG
fliG-F	GCGCGGTACTTTTGATGACC
fliG-R	AAACTCGCCCAATACCTCGG
fliD-F	TTTCATCACTGGGGATCGGC
fliD-R	CTTGGCCTTGTAGCTGCTCT
fliN-F	CCGGTCAGAATAACGGCGAT
fliN-R	TGATGGTCATCTTGGTGCGG
fliF-F	AAACAGCCGAGCAATGAGGA
fliF-R	ATCCATTGGCGGATCACCAG
fliT-F	GCACAGCCAAACTGATGTCC
fliT-R	TGGCGTAGCTCATCCATTCG
flhB-F	CATGTTCTCCAGCCAGGTGT
flhB-R	ACATTCCCACCAGATGCAGG
flgG-F	CTGTACCAAACCCTGCGTCA
flgG-R	CCTTGCTGTTGTTGGTCTGC
flgH-F	TATCCTGACCATCGTGCTGC
flgH-R	GTTATCACCGTTGGCCTCCA
flgI-F	CGCAACGTCGAACTGGATTC
flgI-R	ATCTGGGTATTCGGCGTCAC

flhA-F	CACAGATCCAGGGCTACACC
flhA-R	CTCCTGAGAGACCCGATCCA
fliH-F	GGGTTATGACGCGGGTTTTC
fliH-R	CGGCAATGACGCTATCAAGG
fliA-F	GACGAATGGCGTGAGGATCA
fliA-R	GTCAGCACCAGCTTTTCACG
flgE-F	ACCTCGATGACCACCTACGA
flgE-R	CGCTGTCGATGGAGTAGACC
β-actin-F	ATGGATGAGGAAATCGCTGCC
β-actin-R	CTCCCTGATGTCTGGGTCGTC
il-8-F	GTCGCTGCATTGAAACAGAA
il-8-R	CTTAACCCATGGAGCAGAGG
il-10-F	TCACGTCATGAACGAGATCC
il-10-R	CCTCTTGCATTTCACCATATCC
il-15-F	ACAGAGGAAGAAGCCTACAG
il-15-R	GCGATGAAGACGAGAAAGAG
il-1β-F	TGGACTTCGCAGCACAAAATG
il-1β-R	GTTCACTTCACGCTCTTGGATG
ifn-F	GAATGGCTTGGCCGATACAGGATA
ifn-R	TCCTCCACCTTTGACTTGTCCATC
tnf-α-F	TGTGCGTTGAAGATGTTGAA
tnf-α-R	AGGTAAATGGTGCTGTAGGA
lyz-F	GATTTGAGGGATTCTCCATTGG
lyz-R	CCGTAGTCCTTCCCCGTATCA
CD4-1-F	CATTTACGCACAGGTAGGAG
CD4-1-R	CTGCGGGTTCCTGTTGATTG
MHC IIβ-F	CTCTGCTGGCTGGGATACTG
MHC IIβ-R	GGGCTCGCATGGCAGATTTA
gyrB-F	AAACTGGTGGCGGAGCATTA

gyrB-R	CGCTAAGGGTCGGCTGATAG
pMW82-GFP-F	CTGTCCACACAATCTGCCCT
pMW82-GFP-R	TGCCATGTGTAATCCCAGCA
